# DSC-Net: Enhancing Blind Road Semantic Segmentation with Visual Sensor Using a Dual-Branch Swin-CNN Architecture

**DOI:** 10.3390/s24186075

**Published:** 2024-09-20

**Authors:** Ying Yuan, Yu Du, Yan Ma, Hejun Lv

**Affiliations:** Beijing Key Laboratory of Information Service Engineering, College of Robotics, Beijing Union University, Beijing 100101, China; 20221083510910@buu.edu.cn (Y.Y.); jqrmayan@buu.edu.cn (Y.M.); lyuhejun@163.com (H.L.)

**Keywords:** semantic segmentation, transformer, blind roads segmentation, edge information, visual sensors

## Abstract

In modern urban environments, visual sensors are crucial for enhancing the functionality of navigation systems, particularly for devices designed for visually impaired individuals. The high-resolution images captured by these sensors form the basis for understanding the surrounding environment and identifying key landmarks. However, the core challenge in the semantic segmentation of blind roads lies in the effective extraction of global context and edge features. Most existing methods rely on Convolutional Neural Networks (CNNs), whose inherent inductive biases limit their ability to capture global context and accurately detect discontinuous features such as gaps and obstructions in blind roads. To overcome these limitations, we introduce Dual-Branch Swin-CNN Net(DSC-Net), a new method that integrates the global modeling capabilities of the Swin-Transformer with the CNN-based U-Net architecture. This combination allows for the hierarchical extraction of both fine and coarse features. First, the Spatial Blending Module (SBM) mitigates blurring of target information caused by object occlusion to enhance accuracy. The hybrid attention module (HAM), embedded within the Inverted Residual Module (IRM), sharpens the detection of blind road boundaries, while the IRM improves the speed of network processing. In tests on a specialized dataset designed for blind road semantic segmentation in real-world scenarios, our method achieved an impressive mIoU of 97.72%. Additionally, it demonstrated exceptional performance on other public datasets.

## 1. Introduction

In navigation systems for the visually impaired, various sensors are available to provide navigational assistance. Among them, ultrasonic and infrared sensors offer close-range obstacle detection, while LiDAR can create precise long-range 3D environmental maps. Geomagnetic sensors and GPSs guide users in wide outdoor environments, helping to maintain the navigation path. Despite the unique strengths of each sensor, visual sensors have emerged as the core technology due to their low cost and the rich environmental information they provide. By capturing complex visual data, visual sensors enable advanced computer vision functions, such as object recognition [1] and scene analysis, which are challenging for other sensors to achieve.

When integrated with deep learning techniques, visual sensors not only enhance the accuracy and responsiveness of navigation, but also maintain stable performance under varying lighting and weather conditions, significantly improving the overall performance and reliability of the system. In navigation assistance systems for the visually impaired, visual sensors and image segmentation technology play a crucial role [2]. Visual sensors capture real-time image data from the environment, which are then categorized into specific classes (such as sidewalks, vehicles, and pedestrians) through semantic segmentation. This process provides visually impaired users with detailed and critical information about their surroundings, greatly enhancing their spatial awareness and directly impacting safe navigation and decision-making.

High-precision semantic segmentation methods play a vital role in recognizing blind roads, especially in camera-assisted technologies designed for the visually impaired. These methods work by accurately identifying pixel categories in images, even under challenging conditions such as varying lighting, weather changes, or occlusions. Image sensors capture the necessary data, producing high-resolution images that serve as input for these segmentation methods. By enhancing the accuracy of environmental interpretation, these methods reduce the risk of misclassification and, consequently, improve the safety of path planning. Furthermore, when the navigation system can consistently provide reliable information, user trust and confidence in the technology are significantly boosted. This precision and dependability also enable the system to adapt effectively to complex and changing environmental conditions, ensuring stable operation in all situations. Ultimately, this not only increases the acceptance and frequency of technology use but also greatly enhances the daily quality of life and independence of visually impaired users.

Recently, prominent Convolutional Neural Networks (CNNs) such as Fully Convolutional Networks (FCNs) [3], U-Net [4], and their variants have significantly advanced semantic segmentation, demonstrating enhanced performance [5,6]. These models, especially those with an encoder–decoder structure, effectively capture high-level semantics. The encoder processes global context through convolution and pooling operations, while the decoder rebuilds the feature maps for pixel-level semantic prediction via upsampling and skip connections. Models like SegNet [7], PSPNet [8], and HRNet [9] utilize this architecture. SegNet [7] restores image information through its decoder but lacks a robust mechanism for integrating multi-scale contextual information. PSPNet [8] captures global contextual information at multiple scales through its pyramid pooling module but demands high computational resources. HRNet [9] improves high-level semantic feature representation by connecting convolutional streams at different resolutions in parallel, thereby enhancing resource efficiency.

**Figure 1:**
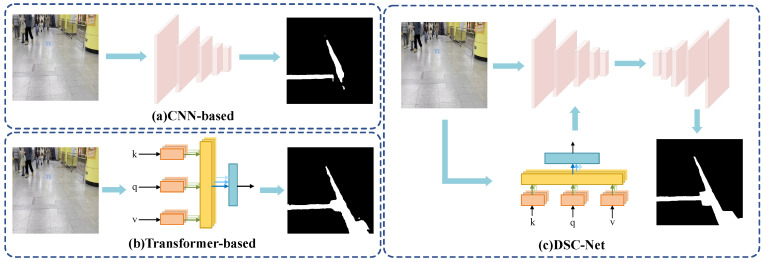
(**a**) CNN-based methods excel at handling detailed information but struggle to capture long-range dependencies. They have difficulty understanding context when external conditions change significantly. (**b**) In contrast, transformer-based methods lead to unclear edge information in the results. (**c**) DSC-Net includes both a CNN-based branch and a transformer-based branch. This design effectively addresses both context and edge details.

In complex environments, blind roads often face issues such as occlusions and truncations, which lead to the loss of detail and blurred edge segmentation, posing significant challenges (see Figure 1a). Various methods have been developed to aggregate global context from local details through multi-scale or multi-branch fusion [10,11]. Yuan et al. [12] introduced a feature pyramid branch to enhance feature map fusion across different levels. FPANet [13] utilizes a semantic bidirectional feature pyramid network (SeBiFPN) to integrate semantic and spatial information effectively, enhancing features from different levels. DDRNet [14] achieves the efficient fusion of detail and semantic information through multiple bi-directional multi-scale connections between its two branches. Additionally, some methods leverage self-attention mechanisms to aid CNNs in feature extraction, applying self-attention to feature maps generated by the CNN backbone to boost the encoder’s ability to represent long-range spatial dependencies [15,16]. However, due to the inherent biases of CNNs, which include locality and translation invariance, these methods struggle with discontinuous individual differences, making it challenging to address variations in type, size, and texture. This necessitates the integration of richer global context information with detailed spatial features to enhance semantic reasoning capabilities.

Motivated by the major breakthroughs that transformers have brought to natural language processing (NLP) [17], researchers have adapted this technology to the visual domain [18]. The Vision Transformer (ViT) [19], applies the transformer architecture directly to image processing for the first time. It divides an image into patches, incorporates position encoding, and captures dependencies among these patches. ViT has shown exceptional performance on large-scale image datasets like ImageNet, though its extensive computational demands have impacted its processing speed. The Swin-Transformer [20] introduces a hierarchical shifted window approach that significantly reduces computational load and improves global perception via cross-window information exchange. The SETR [21] utilizes a specialized decoder that integrates multi-scale features to optimize information flow and feature utilization. Although transformers are adept at capturing long-range dependencies, they struggle with acquiring detailed information, such as edges, crucial for detecting subtle defects (see Figure 1b). The demonstrated success of ViT [19], Swin-Transformer [20], and SETR [21] across various computer vision tasks underscores the potential of transformers in this field. While Swin-Transformer-based methods have advanced in medical image segmentation [22] and remote sensing image segmentation [23], their application in blind road semantic segmentation has yet to be explored.

Inspired by the advancements of the Swin-Transformer, we developed Dual-Branch Swin-CNN Net(DSC-Net), a model that overcomes the limitations of both CNNs and transformers. DSC-Net effectively balances global contextual data with detailed features, making it particularly suited for blind road applications. This model uses a CNN as the primary encoder and the Swin-Transformer as a secondary encoder. Together, they constitute a parallel dual-encoder configuration resembling U-Net (see Figure 1c). The Spatial Blending Module (SBM) connects two Swin-Transformer blocks to improve the fusion of information. Additionally, the Inverted Residual Module (IRM), which incorporates depthwise separable convolution, reduces the number of parameters and speeds up computation. The hybrid attention module (HAM) within the IRM further refines the extraction of fine details. Our key contributions are as follows:

- We propose a parallel architecture combining CNN and transformer technologies to precisely detect blind roads. We have also created a semantic segmentation dataset for blind roads that includes samples from complex environments.
- The Inverted Residual Module with depthwise separable convolution enhances segmentation speed, while the hybrid attention module optimizes feature representation. The Spatial Blending Module is engineered to improve global information perception.
- Performance tests on the Cityscapes dataset, Blind Roads and Crosswalks dataset, and Blind Roads dataset were conducted to validate the efficacy of our method.

## 2. Related Work

### 2.1. Semantic Segmentation of Blind Roads

In blind road semantic segmentation, both image processing and CNN-based methods demonstrate unique strengths and limitations. Image processing primarily relies on local image attributes, which limits its ability to grasp global information [24,25]. Conversely, CNN-based methods offer considerable advantages. For example, Liu et al. [26] enhanced their network’s feature extraction component by replacing standard convolutions with inverted residual blocks, effectively mitigating the environmental vulnerabilities associated with traditional image processing techniques. Cao et al. [27] created a lightweight network tailored for the segmentation of blind roads and pedestrian crossings, achieving a balance between accuracy and real-time computational performance. Nguyen et al. [28] merged a hierarchical Gaussian process classifier with an encoder–decoder structure. However, its high computational demands limit real-time prediction capabilities. Chen et al. [29] integrated atmospheric transmission and thermal inertia effects into their segmentation tasks, which improved segmentation in low-light conditions, although their model is limited to a single structure. While transformers [17] initially achieved prominence in natural language processing, their application in computer vision [21,30,31] has shown promise, yet they have not significantly influenced blind road segmentation. In this paper, we present DSC-Net, an advanced segmentation network designed for blind roads. By combining the strengths of CNNs and transformers, DSC-Net enhances both segmentation accuracy and robustness, even under complex conditions.

### 2.2. Global Context Information

Extracting contextual information from images in complex environments is essential for understanding pixel relationships and refining object boundaries. Traditional image processing methods perform well in simple settings with clear distinctions but falter in more complex scenes [32]. Some CNN approaches employ Spatial Pyramid Pooling (SPP) [33] and its variants [8,12,34] to capture global information across multiple scales, often at the expense of fine spatial details. Cao et al. [27] improved multi-scale and contextual information using densely connected Atrous Spatial Pyramid Pooling modules. While dilated convolutions broaden the receptive field and enhance broader context capture, they can introduce gridding effects and produce discontinuous features [35]. Integrating CNN methods with self-attention allows for efficient feature combination within specific regions [36,37]. Fu et al. [37] developed recurrent thrifty attention, which selectively processes the most relevant sections of the input data. DANet [38] addresses semantic dependencies separately across spatial and channel dimensions, and AGPCNet [39] utilizes an attention-guided pyramid context mechanism. Although these methods improve global information processing, they restrict global data integration to specific areas. Transformers fully compute relationships between all pixels, yet their high computational demands limit speed. Yang et al. [40] introduced CSwin-PNet, which harnesses transformer advantages to establish long-distance dependencies and acquire comprehensive global context, overcoming CNN limitations. Xu et al. [41] merged visual markers with a progressive sampling visual transformer to effectively manage spatio-temporal context information. Li et al. [42] combined CNNs with transformers and introduced a contextual attention mechanism, enhancing dynamic focusing and allowing global information to adaptively influence local feature processing.

### 2.3. Occlusion Edge Features

In semantic segmentation, occlusion significantly complicates the process. Enhancing edge information extraction can notably improve segmentation performance in occluded conditions. To address feature confusion in occluded remote sensing images, Li et al. [43] introduced an occlusion localization and occlusion-guided multi-task interaction method for accurate occlusion identification. Zheng et al. [44] developed a boundary supplementary mask that processes borderline pixels and reduces clustering errors. In video instance segmentation (VIS), some techniques track occluded objects using frame-by-frame feature embeddings [45] or associations [46]. However, these methods are limited to learning decision boundaries between instances and do not model the underlying spatiotemporal data distribution [47]. Data enhancement techniques can effectively mitigate occlusion issues. Chen et al. [48] proposed a technique that simulates occlusions more realistically by embedding new instances into the image based on contextual information. Ke et al. [49] introduced BCNet, a dual-layer mask prediction network that addresses severe occlusion and overlap in instance segmentation, suitable for two-stage but not single-stage segmentation. The U-Net’s [4] encoder–decoder structure excels at capturing multi-scale image information, enhancing robustness against occlusion. Attention modules within U-Net prioritize critical image regions, significantly improving edge information processing and feature extraction capabilities for occluded objects [50,51]. To address target occlusion in complex scenes, Fu et al. [38] introduced DANet (Dual Attention Network), which utilizes dual attention mechanisms for spatial and channel attention to capture fine-grained information. He et al. [52] designed a spatial interaction structure that enhances edge clarity in occluded conditions. Li et al. [53] proposed a transformer-based encoder–decoder approach for occluded pedestrian re-identification (Re-ID), achieving part discovery under weak supervision.

## 3. Methods

### 3.1. Architecture

In common vision sensor-based navigation systems designed for visually impaired individuals, a parallel dual-encoder structure, comprising both CNN and Swin-Transformer, can significantly enhance system performance. As the primary data acquisition tool, visual sensors capture real-time environmental images, providing the foundational data for semantic segmentation.

**Figure 2:**
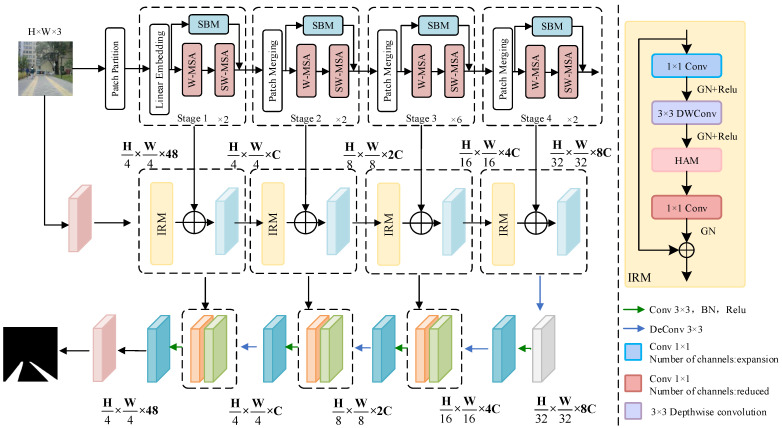
Overview of DSC-Net. An encoder–decoder structure with skip connections is employed, which establishes the relationship between the encoder and decoder. The encoder incorporates a transformer-based global context branch and a CNN-based detail branch, processing images and capturing multi-scale information. These branches are merged and upsampled by the decoder to generate segmentation outputs.

Our method is illustrated in Figure 2. The DSC-Net utilizes a parallel architecture combining a CNN encoder and a Swin-Transformer to balance local feature extraction and global context modeling. The CNN branch focuses on extracting detailed information from the captured images, such as edges and textures, which is crucial for identifying various obstacles and ground types in complex urban environments. Meanwhile, the Swin-Transformer branch handles broader scene information, identifying and distinguishing the expansive spatial layouts, such as the areas separating sidewalks from roadways. This global perspective is essential for planning safe and effective navigation routes.

The method further integrates a Spatial Blending Module (SBM) and a hybrid attention module (HAM) to facilitate deep information fusion between the two processing branches, achieving more coherent and precise environmental analysis. The SBM plays a key role in integrating features from both branches. By blending spatial information across different layers, SBM ensures that global and local features are effectively combined, reducing the impact of occlusions and noise in blind road images. The HAM improves boundary detection by applying attention mechanisms to both spatial and channel dimensions. This ensures that the model can accurately detect road edges, even in challenging conditions where boundaries are blurred. Additionally, an Inverted Residual Module (IRM) is incorporated to accelerate the network’s processing speed, ensuring that the system can respond in real time, thereby meeting the dynamic navigation needs of visually impaired users.

Through integration, the navigation system can provide detailed and accurate environmental information. It can also dynamically adjust navigation instructions based on the complexities of urban environments. It significantly enhances the independent mobility and overall safety of visually impaired individuals.

The network processes input images of size H×W×3. It applies convolution to create overlapping 8 × 8 patch tokens with 50% overlap, which are then flattened and concatenated in a linear embedding layer. This leads to an auxiliary encoder composed of Swin-Transformer modules operating in four stages. Each stage includes patch merging that downsamples the image into the $C_{1}$ dimension, followed by a Swin-Transformer block that processes the features. The output feature maps from these stages are $\frac{H}{4}\times \frac{W}{4}\times 128$, $\frac{H}{8}\times \frac{W}{8}\times 256$, $\frac{H}{16}\times \frac{W}{16}\times 512$, and $\frac{H}{32}\times \frac{W}{32}\times 1024$, respectively, halving the dimensions and doubling the channel count at each subsequent stage. The main encoder contains four inverted residual blocks, each augmented with hybrid attention modules to enhance detailed information extraction. Feature map sizes in the main encoder match those in the auxiliary encoder at corresponding stages. Skip connections between the main and auxiliary encoders enrich the feature extraction with both semantic and detailed information.

During the fourth stage of encoding, a 2 × 2 transposed convolution layer enlarges the feature map to $\frac{H}{32}\times \frac{W}{32}\times 1024$. This map is then transferred to the decoder, which includes three stages of 3 × 3 convolution layers that reduce the feature channel count and 2 × 2 transposed convolutions that increase resolution. Each convolution is immediately followed by the application of batch normalization (BN) and ReLU activation. The resulting feature maps are $\frac{H}{16}\times \frac{W}{16}\times 512$, $\frac{H}{8}\times \frac{W}{8}\times 256$, and $\frac{H}{4}\times \frac{W}{4}\times 128$, corresponding to the third, second, and first stages of the encoder, respectively. The final segmentation output is produced using a 3 × 3 convolution followed by linear interpolation for upsampling.

### 3.2. Spatial Blending Module

Despite using a strategy that alternates between regular and shifted windows, the Swin-Transformer still struggles to maintain strong global modeling capabilities. Additionally, obstructions in blind road images cause blurred boundaries, necessitating the removal of certain spatial information. To improve interaction with global contextual information, we introduced the Spatial Blending Module (SBM). This module incorporates branches that extract spatial information from both convolutional and pooling layers. To capture essential information distributions on the feature map, average pooling is applied in both vertical and horizontal directions. Then, it is reintegrated into the main process of the Swin-Transformer block, enhancing its global modeling capabilities.

**Figure 3:**
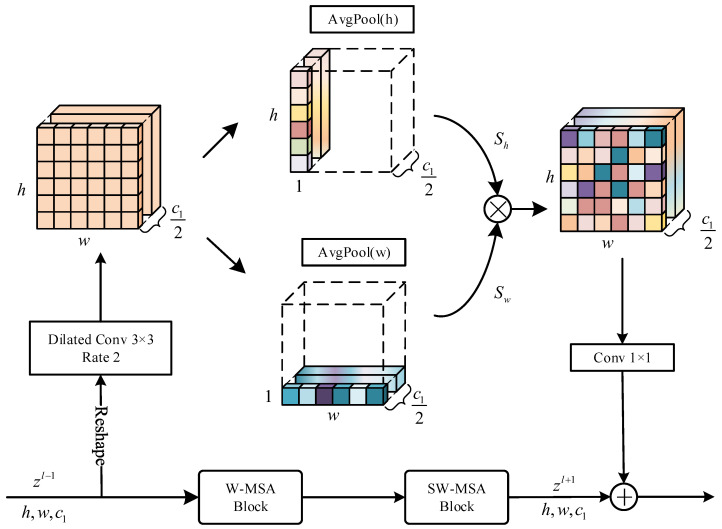
The structure of Spatial Blending Module (SBM). Statistical features are captured along horizontal and vertical directions, reshaped through matrix multiplication. Finally, they are integrated with the input features. SBM can further enhance the interaction of global contextual information.

Figure 3 presents the architecture of the Spatial Blending Module (SBM). For a given auxiliary encoding stage n (n∈1,2,3,4), the input data $z^{\left(l-1\right)}\in R^{\left(h\times w\right)\times c_{1}}$ are first reshaped into $s\in R^{h\times w\times c_{1}}$. Here, $c_{1}=2^{n-1}C_{1}$, $h=\frac{H}{2^{n+1}}$, and $w=\frac{W}{2^{n+1}}$. The feature *s* is then processed by a dilated convolution, utilizing a dilation rate of 2 for a larger receptive field. Meanwhile, the channel count is reduced to $k=\frac{c_{1}}{2}$ to decrease computational load, producing the output feature $\hat{s}$. Subsequently, $\hat{s}$ undergoes average pooling in both vertical and horizontal directions, resulting in vertical features $s_{h}\in R^{h\times 1\times k}$ and horizontal features $s_{w}\in R^{1\times w\times k}$. The computation formulas are as follows:

(1) $$s_{h_{i}}^{k}=\frac{1}{w}\sum_{j=0}^{w-1}{\hat{s}}^{k}\left(i,j\right)$$

(2) $$s_{w_{j}}^{k}=\frac{1}{h}\sum_{i=0}^{h-1}{\hat{s}}^{k}\left(i,j\right)$$

here, *i* represents the vertical direction in space (0≤i<h), *j* represents the horizontal direction (0≤j<w), and *k* denotes the channel count. The feature $s_{h}$ and the feature $s_{w}$ are multiplied element-wise to obtain a position-related attention map *S*, where $S\in R^{h\times w\times k}$. Finally, *S* is processed through a 1 × 1 convolution which includes the GELU activation function and batch normalization. Then, it is added element-wise to the output feature $z^{l+1}$ produced by the SW-MSA stage, resulting in the output feature of SBM. This process is represented as

(3) $$F_{S}=z^{\left(l+1\right)}⊕\phi \left(s_{h}⊗s_{w}\right)$$

where ⊕ denotes element-wise addition, ⊗ denotes matrix multiplication, and ϕ(·) represents a 1 × 1 convolution including GELU activation function and batch normalization.

### 3.3. Inverted Residual Module

To accelerate computation while maintaining high-quality edge feature extraction, we introduce the Inverted Residual Module (IRM). Traditional convolution operations, which process the entire input feature map, are limited by their high computational load. Depthwise separable convolutions reduce computation but may sacrifice some feature details. IRM addresses this by expanding and then reducing the channel count within the residual network, compensating for the loss of details. We use depthwise separable convolutions with weight normalization to lower the computational load while facilitating the network’s ability to discover new features. Additionally, incorporating the hybrid attention module (HAM) improves the emphasis on the edge information of features, which will be further elaborated on in the following section.

**Figure 4:**
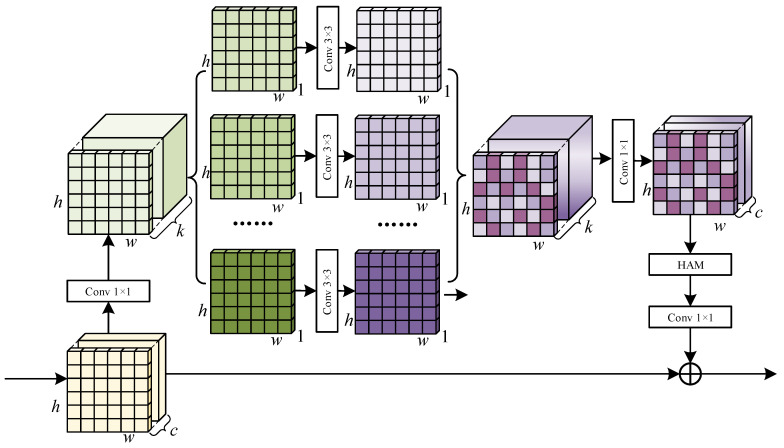
The structure of the Inverted Residual Module (IRM) is designed to accelerate computation speed. The number of image channels is expanded to extract features from each channel. The channel count is then reduced back to the original.

In Figure 4, for stage n, the input feature size of the main encoder is $F\in R^{h\times w\times c}$. Initially, 1 × 1 convolution expands the channel count to k, followed by feature transformation using 3 × 3 depthwise convolution and then 1 × 1 pointwise convolution for weight normalization. The normalization process for the weight $weight\in R^{H\times W\times C_{\mathrm{in}}\times C_{\mathrm{out}}}$ of each output channel $C_{\mathrm{out}}$ with $W_{c}\in R^{C_{\mathrm{in}}\times H\times W}$ is described below:

(4) $$m_{c}=\frac{1}{C_{\mathrm{in}}\times H\times W}\sum_{i,j,k}W_{c}\left(i,j,k\right)$$

(5) $$v_{c}=\frac{1}{C_{\mathrm{in}}\times H\times W}\sum_{i,j,k}{\left(W_{c}\left(i,j,k\right)-m_{c}\right)}^{2}$$

where $m_{c}$ represents the mean of $W_{c}$, and $v_{c}$ denotes its variance. The normalized weight $W_{c}^{\prime }$ is calculated as

(6) $$W_{c}^{\prime }\left(i,j,k\right)=\frac{W_{c}\left(i,j,k\right)-m_{c}}{\sqrt{v_{c}+\epsilon }}$$

where ϵ is set to $1\times 10^{-5}$ for numerical stability. Subsequently, the feature map is processed through HAM, followed by a 1 × 1 convolution, which decreases the channel count to the original size of c. Finally, the processed feature map is element-wise added to *F*, resulting in the IRM output feature $F_{I}$, calculated as

(7) $$F_{I}=\mathrm{GN}\left(C_{2}^{1\times 1}\left(H\left(f_{2}\left(\mathrm{DW}\left(C_{1}^{1\times 1}\left(F\right)\right)\right)\right)\right)\right)+F$$

here, $C_{1}^{1\times 1}$ and $C_{2}^{1\times 1}$ represent 1 × 1 convolutions, DW denotes depthwise separable convolution, $f_{1}\left(\cdot \right)$ and $f_{1}\left(\cdot \right)$ denote GN and ReLU, and H(·) is the computation process of the HAM module.

### 3.4. Hybrid Attention Module

CNNs face challenges in extracting valuable information from feature maps when blind roads are partially obscured. In response to this problem, we propose the hybrid attention module (HAM) that integrates channel and spatial attention, making the network more capable of discerning blurred edges. Channel attention selectively emphasizes essential feature channels, improving the interpretation of complex scenes and clarifying the overall shape of obscured blind roads. Meanwhile, spatial attention focuses on analyzing visible areas, enhancing spatial positioning accuracy around these regions. This dual focus enables a more precise contextual interpretation of the entire scene.

**Figure 5:**
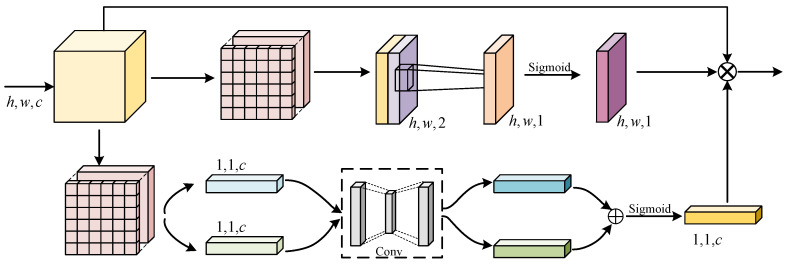
The structure of the hybrid attention module (HAM). Input features are processed through channel and spatial attention branches. Global pooling, multilayer perceptrons, and convolutions extract key channel and spatial features. These features are then integrated with the input features to produce the output features. HAM focuses more on the edge information of occlusions.

As shown in Figure 5, HAM includes both channel attention and spatial attention branches. Assuming that the size of the input feature map is $F\in R^{h\times w\times c}$, after processing through these branches, we obtain the attention map for channel $F_{C}^{\prime }$ and the attention map for spatial $F_{V}^{\prime }$. Multiplying these maps with *F* element-wise results in the output feature $F_{H}$ of HAM. The computation process is represented as

(8) $$F_{H}=F_{C}^{\prime }⊗F_{V}^{\prime }⊗F$$

where ⊗ indicates matrix multiplication.

In the channel attention, global average pooling and max pooling are applied to *F*, resulting in two channel-wise statistical measures, $F_{\mathrm{Avg}}^{C}\in R^{1\times 1\times c}$ and $F_{\mathrm{Max}}^{C}\in R^{1\times 1\times c}$. These measures pass through two depthwise separable convolutions, and the results are added and processed with a sigmoid function, resulting in the attention map for channel $F_{C}^{\prime }$. The following formula represents it:

(9) $$F_{C}^{\prime }=\mathrm{sigmoid}\left(\mathrm{DW}(\mathrm{AvgPool}(F))+\mathrm{MLP}(\mathrm{DW}(F))\right)$$

where AvgPool represents global average pooling, MaxPool denotes max pooling. DW denotes depthwise separable convolution, and sigmoid denotes the sigmoid activation function.

In the spatial attention branch, the input feature *F* is averaged and max pooled, then concatenated to achieve dimensions h×w×2. This feature is processed by a convolution to produce the attention map for spatial $F_{V}^{\prime }$, calculated as

(10) $$F_{V}^{\prime }=\mathrm{sigmoid}\left(\mathrm{Conv}\left([\mathrm{AvgPool}(F);\mathrm{MaxPool}(F)]\right)\right)$$

where Conv denotes the convolution operation.

## 4. Experiments

Evaluations were carried out to confirm the effectiveness and generalizability of DSC-Net on multiple well-known datasets, including one specifically focused on blind roads. The Cityscapes dataset, known for its complex urban street scenes, provides an excellent benchmark for estimating the performance of semantic segmentation networks. Additionally, the Blind Roads and Crosswalks dataset, which focuses on blind roads and pedestrian crosswalks, requires high precision in recognition and segmentation, despite its visual simplicity. We tested DSC-Net’s performance and generalizability across the Cityscapes, Blind Roads and Crosswalks, and Blind Roads datasets. Furthermore, we specifically assessed the performance of individual modules within DSC-Net on the Blind Roads dataset.

### 4.1. Datasets

**Cityscapes Dataset**. It contains approximately 5000 high-quality urban scene images, each with a resolution of 1024 × 2048 pixels. It provides pixel-level semantic segmentation labels for 19 different categories, divided into a training set of 2975 images, a validation set of 500 images, and a test set of 1525 images. This dataset, which extensively covers urban environments, offers detailed pixel-level semantic labels, making it an ideal foundation for optimizing and testing semantic segmentation models in navigation systems designed for visually impaired individuals.

Moreover, the diverse range of urban scenes, including various city structures and environmental changes, makes Cityscapes crucial for training models to adapt to the varied conditions encountered in urban navigation. Additionally, due to its widespread recognition in both academic and industrial circles, as well as its status as a performance benchmark, using this dataset to demonstrate our method not only enhances the credibility of our research but also facilitates fair comparison with other state-of-the-art technologies, thereby validating the effectiveness and superiority of our approach.

**Blind Road and Crosswalk Dataset**. It is specifically designed to address critical elements like blind roads and crosswalks in navigation systems for visually impaired individuals. It includes 200 images collected under real-world conditions, each with a resolution of 512 × 512 pixels. These images are all semantically labeled, with the dataset divided into 150 images for training, 25 images for validation, and 25 images for testing. The design of this dataset directly corresponds to practical application needs, ensuring that the training objectives are closely aligned with the navigation requirements of visually impaired users, thereby enabling the trained models to more accurately recognize these crucial navigation markers.

Consequently, the use of the Blind Roads and Crosswalks dataset not only enhances the practicality and reliability of the system but also, due to its high relevance to specific scenarios, makes it an important resource for evaluating and optimizing navigation technologies for the visually impaired.

**Blind Roads Dataset**. It is a custom-built dataset specifically focused on the characteristics of blind roads. It provides realistic scene information by capturing images from a fixed height and horizontal viewpoint using a handheld camera, taking into account environmental factors. The dataset contains 2300 images of blind roads, each with a resolution of 512 × 512 pixels, divided into a training set of 1534 images, and two validation and test sets, each containing 383 images. All images are equipped with precise semantic labels for blind roads.

This dataset not only includes a large number of blind road images but also places special emphasis on the annotation quality of each image, ensuring a high degree of accuracy in the data. Such a high-standard dataset is crucial for training models that can accurately recognize various blind road features, especially in applications where it is essential to ensure that visually impaired individuals can safely navigate blind roads.

The Blind Roads dataset not only fills the gap in the availability of high-quality blind road data but also ensures data precision through its rigorous annotation standards.

### 4.2. Implementation

**Training Setup**. To ensure fairness in the training process and comparability of the results, the experimental setup is detailed in Table 1, and the training parameters are listed in Table 2. The experimental setup was configured as follows: the operating system was Ubuntu 22.04, with an Intel Xeon Gold 6326 processor (Intel, Santa Clara, CA, USA), an NVIDIA Tesla V100 GPU (NVIDIA, Santa Clara, CA, USA), and 32 GB of RAM. The network was developed with PyTorch1.12.1, Python 3.8, leveraging CUDA 11.7. The Adam optimizer was employed, starting with a learning rate of 0.001. The training process used a batch size of 8, input images of 512 × 512 pixels, and a total of 100 training epochs.

**Loss Functions**. We employ a combination of Dice Loss and Binary Cross-Entropy (BCE) Loss as our loss function. Dice Loss primarily enhances the model’s performance in edge segmentation tasks, while BCE Loss excels in distinguishing between two classes. The formulas for these loss functions are as follows:

(11) $$L_{\mathrm{Dice}}=1-\frac{2\sum_{i=1}^{N}y_{i}\hat{y_{i}}+1}{\sum {i=1}^{N}y_{i}^{2}+\sum_{i=1}^{N}{\hat{y}}_{i}^{2}+1}$$

(12) $$L_{\mathrm{BCE}}=-\frac{1}{N}\sum_{i=1}^{N}\left[y_{i}\log\left({\hat{y}}_{i}\right)+\left(1-y_{i}\right)\log\left(1-{\hat{y}}_{i}\right)\right]$$

where $y_{i}$ denotes the actual label value of pixel *i*, $\hat{y}i$ represents the predicted value for pixel *i*, and *N* stands for the total number of pixels. The final loss function is a weighted combination of Dice Loss and BCE Loss, defined as:

(13) $$L=\alpha L_{\mathrm{Dice}}+\beta L_{\mathrm{BCE}}$$

where α and β are the weight factors for their respective loss functions. In this experiment, α was set to 0.7 and β was 0.3.

**Evaluation Metrics**. Mean Intersection over Union (mIoU) and F1-score were chosen as the evaluation metrics for assessing the performance of semantic segmentation tasks. MIoU is calculated as the average of the IoU values for all categories, with IoU defined as:

(14) $$\mathrm{IoU}=\frac{\mathrm{TP}}{\mathrm{TP}+\mathrm{FP}+\mathrm{FN}}$$

where TP stands for true positives, FP for false positives, and FN for false negatives. The formula for the F1-score is:

(15) $$F1\text{-}\mathrm{score}=2\times \left(\frac{\mathrm{Precision}\times \mathrm{Recall}}{\mathrm{Precision}+\mathrm{Recall}}\right)$$

(16) $$\mathrm{Precision}=\frac{\mathrm{TP}}{\mathrm{TP}+\mathrm{FP}}$$

(17) $$\mathrm{Recall}=\frac{\mathrm{TP}}{\mathrm{TP}+\mathrm{FN}}$$

## 5. Experimental Results and Analysis

### 5.1. Comparative Experiment

To validate DSC-Net’s enhanced capabilities in detail-oriented edge extraction and generalization, we conducted experiments on the Cityscapes, Blind Roads and Crosswalks, and Blind Roads dataset. We compared DSC-Net with existing models, including U-Net, FPN, Deeplabv3, Bisenetv1, Vision Transformer(ViT), and Swin-Transformer. While U-Net, FPN, Deeplabv3, and Bisenetv1 are traditional CNN methods, ViT and Swin-Transformer represent classic vision transformer models. Additionally, DSC-Net has an encoder–decoder structure resembling U-Net. We evaluated the networks using metrics such as mIoU, F1-score, Params, and FPS.

#### 5.1.1. Cityscapes Dataset

Aiming to balance the deficiencies of CNN and transformer in capturing global context and detail, we conducted experiments on the Cityscapes dataset. Table 3 shows the performance metrics across various networks. DSC-Net combines the U-Net architecture, CNN’s local information processing capabilities, and Swin-Transformer’s global information processing strengths, enhancing model performance in urban environments. In terms of performance assessment, DSC-Net achieves an mIoU of 76.31% and an F1-score of 83.20, significantly outperforming the classic encoder–decoder architecture of U-Net. As a benchmark semantic segmentation network, U-Net is highly regarded for its precise capture of local features and effective information transfer mechanisms, with its skip connections significantly aiding in detail recovery. The enhanced capability of DSC-Net was mainly attributed to its optimized design for handling both local and global information. Additionally, Bisenetv1 uses a dual-path convolutional network design, one path processing local information quickly and the other focusing on global context; Deeplabv3 enhances the capture of global information through spatial pyramid pooling of features at different scales. DSC-Net, combining the dual-branch structure of spatial pyramids and Swin-Transformer, surpasses Bisenetv1 and Deeplabv3 in accuracy, particularly in handling complex urban scenes. The Vision Transformer (ViT) divides images into patches and employs a transformer to capture global features, combined with a decoder to restore resolution. ViT-base achieves an mIoU of 71.47% with 142 M parameters and 7.33 FPS, while ViT-large achieves an mIoU of 73.12% with 307 M parameters and 5.23 FPS. As the ViT-base model has a larger parameter count than our method on the Cityscapes dataset, and its performance is inferior to the ViT-large model, we decided to exclude it from further comparisons. The large parameter count and computational complexity of ViT models lead to substantial resource consumption and reduced real-time processing performance. Compared to other transformer-based methods, such as Swin-Transformer and TransUnet, DSC-Net excelled in processing global information. It also captured detailed local features more effectively. This capability is crucial for precise edge information processing.

**Figure 6:**
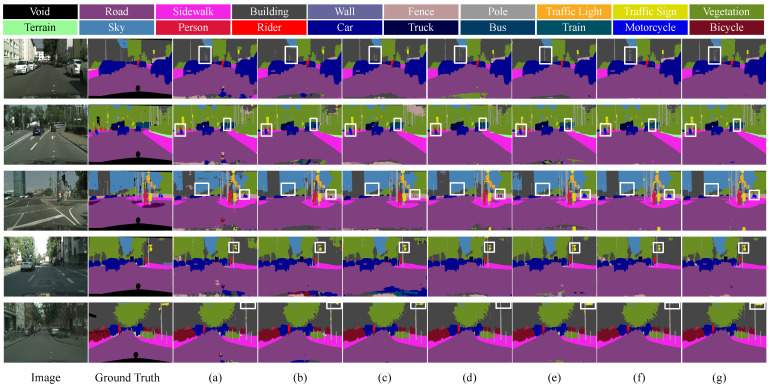
Comparison of semantic segmentation results from Cityscapes dataset. (**a**) U-Net. (**b**) Bisenetv1. (**c**) Deeplabv3. (**d**) Swin-Transformer. (**e**) TransUnet. (**f**) ViT. (**g**) DSC-Net. The rectangles highlight areas where our approach exhibits superior performance. DSC-Net delivers enhanced edge precision for objects including poles, traffic signs, and motorcycles.

**Figure 7:**
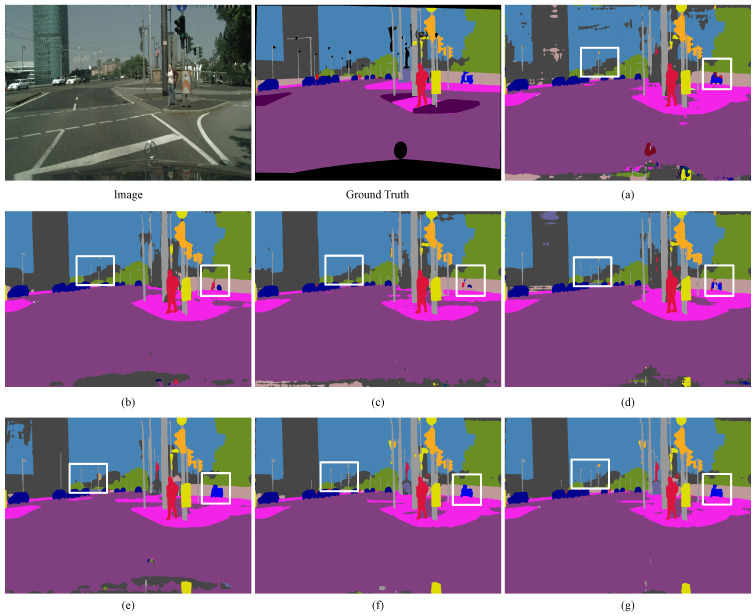
Comparison of an enlarged view of the results from the Cityscapes dataset. (**a**) U-Net. (**b**) Bisenetv1. (**c**) Deeplabv3. (**d**) Swin-Transformer. (**e**) TransUnet. (**f**) ViT. (**g**) DSC-Net.

Figure 6 provides a visual comparison of various methods on the Cityscapes dataset. It clearly demonstrates that our network excels in segmentation performance. In the first row, our network is able to segment distant lampposts more completely, which indicates that it can effectively handle detailed information. In the second and third rows, our network shows a more precise segmentation of motorcycles, signposts, and thin poles. To present a clearer comparison of the key areas, Figure 7 provides an enlarged view of the results from the third row, further emphasizing the distinctions. It performs well when someone is riding a motorcycle, indicating its effectiveness in context understanding during occlusions. This magnified illustration clearly demonstrates that our network exhibits a significant advantage in segmentation performance. The fourth and fifth rows confirm that our network also excels in accurately segmenting traffic signs with specific shapes.

#### 5.1.2. Blind Roads and Crosswalks Dataset

To evaluate DSC-Net’s adaptability and precision in specific applications, we analyzed its performance on the Blind Roads and Crosswalks dataset. This assessment also included the capability to process and reconstruct key information in multiple settings, comparing DSC-Net with other methods.

As shown in Table 4, DSC-Net performs exceptionally well, achieving an mIoU of 94.54% and an F1-score of 97.07 on the Blind Roads and Crosswalks dataset. This dataset focuses on identifying key elements related to visual assistance technologies, such as blind roads and pedestrian crosswalks. Although these elements are visually simple, they require high precision in detection and segmentation. DSC-Net’s outstanding performance on this dataset highlights its high adaptability and precision in specific application scenarios. It is because DSC-Net combines the local information processing power of CNNs with the global semantic processing ability of the Swin-Transformer.

DSC-Net enhances the performance in complex and multi-element scenes. It also maintains efficiency in high-precision segmentation tasks. This integration of technologies enhances the efficiency of information flow. Furthermore, it also improves the capacity to comprehend and reconstruct key information in complex environments.

The diverse complexity of Cityscapes tested the adaptability of DSC-Net in a wide range of environments, while the Blind Roads and Crosswalks Dataset focused on executing specific tasks. DSC-Net maintains high performance on both types of dataset, validating its generalization capability. This generalization demonstrates that DSC-Net is not only suitable for broad urban scenarios but also applicable to specific needs such as road safety and navigation assistance systems.

**Figure 8:**
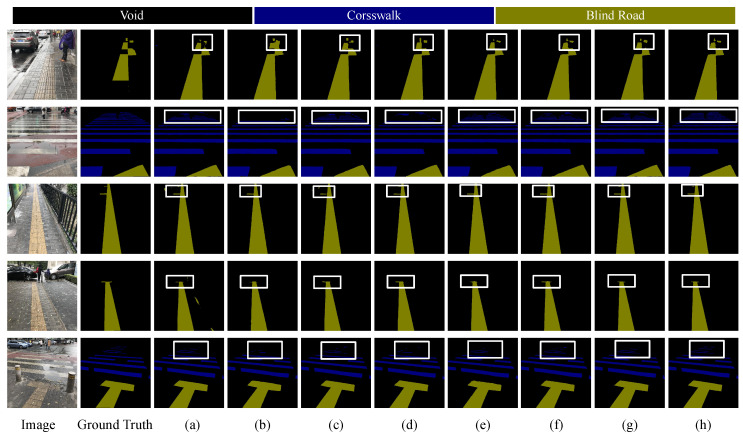
Comparison of semantic segmentation results from Blind Roads and Crosswalks dataset. (**a**) U-Net. (**b**) Bisenetv1. (**c**) SEM_FPN. (**d**) Deeplabv3. (**e**) Swin-Transformer. (**f**) TransUnet. (**g**) ViT-large. (**h**) DSC-Net. The rectangles highlight areas where our approach exhibits superior performance. DSC-Net precisely discerns horizontal blind roads and crosswalks. Additionally, it demonstrates enhanced accuracy on discontinuous vertical blind roads.

The visualization results on the Blind Roads and Crosswalks dataset, as shown in Figure 8, indicate that all networks perform well in processing longitudinal blind paths and can accurately handle nearby transverse blind paths and pedestrian crosswalks. However, at slightly longer distances, the performance of all networks in processing transverse roads declines.

**Figure 9:**
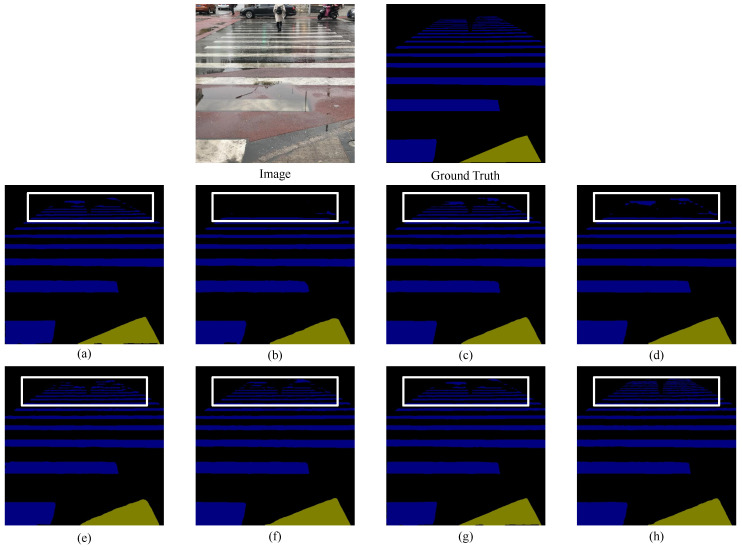
Comparison of an enlarged view of the results from the Blind Roads and Crosswalks dataset. (**a**) U-Net. (**b**) Bisenetv1. (**c**) SEM_FPN. (**d**) Deeplabv3. (**e**) Swin-Transformer. (**f**) TransUnet. (**g**) ViT. (**h**) DSC-Net.

Figure 9 provides a larger view of the second row of results in Figure 8. In Figure 9, due to perspective issues, Bisenetv1 and Deeplabv3 struggle to segment distant transverse pedestrian crosswalks with pedestrian obstructions, whereas U-Net, SEM_FPN, ViT, Swin-Transformer, and TransUnet can roughly outline the shapes but not perfectly. Our network is able to segment the parallel, intermittently continuous shapes of pedestrian crosswalks more completely.

#### 5.1.3. Blind Roads Dataset

Table 5 shows the results on the Blind Roads dataset. It is evident that, in scenarios with fewer categories, all methods perform exceptionally well; yet, our method stands out with its accuracy, achieving, at least, a 1.55% higher mIoU and, at least, 1.05 higher F1-score than any other method. Even though it has the most parameters and operates the slowest among all networks, it still meets the real-time requirements. Among networks capable of real-time performance, our network is 5.81% more accurate than the fastest, Bisenetv1. Despite some sacrifices in speed, our model significantly surpasses other comparative methods in accuracy and robustness, which is crucial for practical applications.

**Figure 10:**
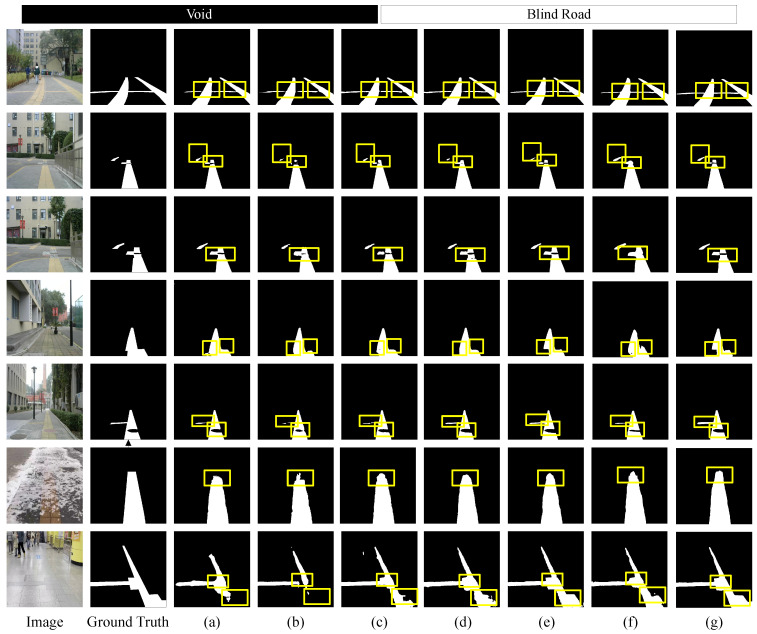
Comparison of semantic segmentation results from Blind Roads dataset. (**a**) U-Net. (**b**) Bisenetv1. (**c**) Deeplabv3. (**d**) Swin-Transformer. (**e**) TransUnet. (**f**) ViT. (**g**) DSC-Net. The rectangles highlight areas where our approach exhibits superior performance. DSC-Net sustains improved contextual relationships on discontinuous blind roads and delivers more distinct edges in the presence of obstructions.

**Figure 11:**
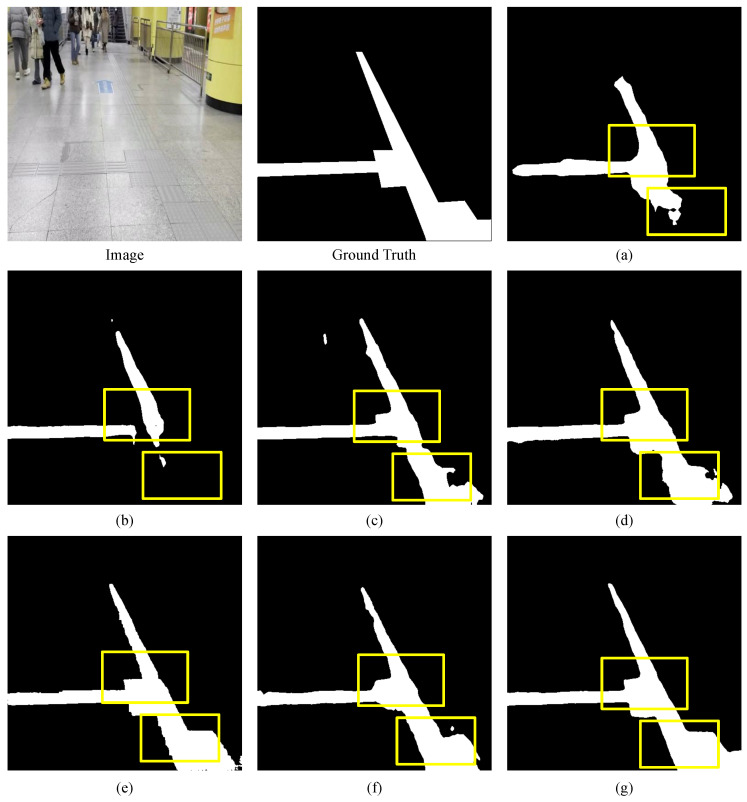
Comparison of an enlarged view of the results from the Blind Roads dataset. (**a**) U-Net. (**b**) Bisenetv1. (**c**) Deeplabv3. (**d**) Swin-Transformer. (**e**) TransUnet. (**f**) ViT. (**g**) DSC-Net.

The visualization results of various networks on the Blind Roads dataset, as shown in Figure 10, indicate that all networks effectively segment blind paths against complex backgrounds, especially the longitudinal paths. However, there are challenges in completely segmenting continuous paths, as illustrated in the first and second rows. Furthermore, our network performs best in segmenting discontinuous, distant paths. The fourth row demonstrates that CNN methods struggle to distinguish between the edges of blind paths and their obstructions, while networks combining transformer and a CNN exhibit the best performance. In the fifth row, compared to other networks, our network better captures the lateral paths and their edge features, achieving superior segmentation results, where others struggle to segment these features. The sixth row present the results under snowy conditions. When the blind road is heavily covered by snow, DSC-Net can still accurately identify the boundaries and structure of the path, demonstrating strong robustness. This suggests that DSC-Net achieves high segmentation accuracy, even under harsh weather conditions, and effectively handles occlusion challenges in complex scenes. In low-contrast environments, such as subway stations, the contrast of visual information is significantly reduced, causing object edges to blend into the background, making clear distinction difficult. This further demonstrates DSC-Net’s adaptability in handling complex lighting and color conditions. As shown in Figure 11, in these situations, CNN-based methods often exhibit instability, whereas transformer-based methods demonstrate greater robustness and adaptability. In particular, DSC-Net, with its multi-scale feature extraction and global context awareness, effectively captures critical details in the scene and accurately segments object boundaries, maintaining high segmentation accuracy. Furthermore, it further highlights DSC-Net’s adaptability in handling complex lighting and color conditions. Overall, while no single model excels in all metrics, our method demonstrates significant advantages in overall performance, particularly suited for the semantic segmentation of blind roads.

### 5.2. Module Effectiveness

#### 5.2.1. Module Ablation

To validate the effectiveness of DSC-Net in details and balancing global information for CNN and transformer, ablation studies were conducted on the Blind Roads dataset. These experiments focused on adaptability and segmentation accuracy in specific applications.

As shown in Table 6, we first studied the performance of TransUnet (baseline). The second group added the SBM to TransUnet, which extracts richer high-level semantic and contextual information and conducts global attention interaction, improving the edge feature extraction. The IoU on blind path categories increased by 0.96%, with mIoU and F1-score increasing by 0.48 and 0.32, respectively. The third group included the IRM, achieving the highest inference speed but sacrificed some accuracy. The fourth group added the HAM, focusing the network more on occluded objects and their edge information, leading to a 1.19% increase in IoU for blind path categories, with mIoU and F1-score improving by 0.64% and 0.33, respectively. Groups five and six added IRM to both SBM and HAM, respectively, compared to the original groups, precision decreased, but FPS significantly increased, achieving a delicate balance between precision and speed without substantially compromising accuracy. From group seven, it was evident that under the combined effect of SBM and HAM, the network’s accuracy was the best, with mIoU reaching 97.78% and F1-score 98.86, though FPS was slightly lower. With the addition of IRM, the loss in accuracy was no more than 0.05%, but inference speed increased by 3 FPS.

#### 5.2.2. Loss Function Weight Ablation

The loss function comprised a weighted combination of Dice Loss and BCE Loss. To achieve better segmentation performance, experiments with different weights were conducted for both loss functions. The evaluation metrics used were mIoU and F1-score. The values of the weights, α and β, correspond to Dice Loss and BCE Loss, with the condition α+β=1.

From Table 7, it is clear that α and β, at different weight values, resulted in significant differences in mIoU and F1-score effects. When α was 0.9 and β was 0.1, the mIoU and F1-score were the lowest, at 97.09% and 98.51, respectively. When α was set to 0.7 and β to 0.3, the mIoU and F1-score reached their maximum values of 97.72% and 98.83, respectively, indicating better segmentation effects. Therefore, we set the weights of α and β to 0.7 and 0.3, respectively.

## 6. Conclusions

In this paper, we introduced DSC-Net, a novel encoder-decoder network integrating transformer and CNN architectures for the semantic segmentation of blind road images. The encoder of DSC-Net consists of two parallel branches: one uses the Swin-Transformer to capture high-level semantic information, while the other employs a CNN to capture low-level detailed information. This design improves computational efficiency while ensuring comprehensive information extraction, particularly excelling in complex scenarios that require an understanding of both global context and local details.

Blind road images captured by camera sensors provided the primary data for our model. By merging high-level semantic information with low-level details, DSC-Net gains multi-granularity features, enhancing representational power. The high-level branch incorporates a Spatial Blending Module to improve global information modeling, while the low-level branch employs an Inverted Residual Module with depthwise separable convolutions to boost computational efficiency. Furthermore, we designed a hybrid attention module to ensure segmentation accuracy and adopted a joint loss function to optimize model performance.

Experimental results demonstrated that DSC-Net exhibited competitive performance on the Cityscapes, Blind Roads and Crosswalks, and Blind Roads datasets. Results from the Blind Roads dataset showed that DSC-Net effectively balances inference speed and accuracy in the blind road segmentation task. DSC-Net performs robustly in environments with partial occlusion and low contrast, owing to its ability to efficiently integrate and process multi-level information in response to varying visual characteristics. In particular, on the Cityscapes dataset, the model’s optimized structure allows for the better capture of complex traffic signs and distant object details, showcasing its adaptability and precision in complex urban scenes.

The advantages of DSC-Net are especially evident in real-world applications. In blind road navigation scenarios, it can assist visually impaired individuals by more accurately identifying the edges of the tactile paths and detecting obstacles, significantly enhancing their safety and autonomy in complex environments. Future research will further explore the integration of multi-source sensor data to advance semantic segmentation technology in blind road navigation.

## Figures and Tables

**Table 1:** Experimental setup.

Environment	Version
Operating System	Ubuntu 22.04
CPU	Intel Xeon Gold 6326
GPU	NVIDIA Tesla V100 32 GB
Compiling Environment	Python 3.8
CUDA	11.7
Deep Learning Framework	Pytorch 1.12.1

**Table 2:** Training parameters.

Parameter	Value
Batch Size	8
Init Learning Rate	0.001
Min Learning Rate	$1\times 10^{-6}$
Image Size	512 × 512
Optimizer	Adam
Epoch	100

**Table 3:** Evaluation of segmentation results of Cityscapes dataset.

Method	mIoU (%)	F1-Score	Params	FPS
U-Net [4]	63.88	71.06	28.99 M	4.97
Bisenetv1 [54]	67.42	79.18	**13.27 M**	**48.31**
Deeplabv3 [35]	65.87	77.75	65.74 M	2.72
Swin-Transformer [20]	73.27	80.83	58.94 M	9.39
TransUnet [55]	74.84	81.54	100.44 M	7.78
Vit-base [19]	71.47	78.16	142 M	7.33
Vit-large [19]	73.12	80.28	307 M	5.23
DSC-Net	**76.31**	**83.20**	133.08 M	7.02

The bold numbers indicate the best values for this metric.

**Table 4:** Evaluation of segmentation results of Blind Roads and Crosswalks dataset.

Method	mIoU (%)	F1-Score	Params	FPS
U-Net [4]	93.19	96.69	28.99 M	46.40
Bisenetv1 [54]	89.81	95.48	**13.27 M**	**134.66**
SE M_FPN [56]	92.47	96.34	28.49 M	69.37
Deeplabv3 [57]	92.21	95.85	65.74 M	37.93
Swin-Transformer [20]	93.31	96.64	58.94 M	26.11
TransUnet [55]	93.62	96.80	100.44 M	22.26
Vit [19]	93.75	96.87	307 M	13.64
DSC-Net	**94.54**	**97.07**	133.08 M	20.29

The bold numbers indicate the best values for this metric.

**Table 5:** Evaluation of segmentation results of Blind Roads dataset.

Method	mIoU (%)	F1-Score	Params	FPS
U-Net [4]	95.18	97.22	28.99 M	47.14
Bisenetv1 [54]	91.91	95.46	**13.27 M**	**115.55**
Deeplabv3 [57]	94.97	96.91	65.74 M	36.53
Swin-Transformer [20]	95.68	97.53	58.94 M	26.13
TransUnet [55]	96.17	97.78	100.44 M	21.81
Vit-large [19]	97.33	98.36	307 M	12.33
DSC-Net	**97.72**	**98.83**	133.08 M	19.59

The bold numbers indicate the best values for this metric.

**Table 6:** Ablation study on the proposed modules with the Blind Roads dataset.

Method	Module			IoU (%)	mIoU (%)	F1-Score	FPS
	SBM	IRM	HAM				
TransUnet				93.54	96.54	98.22	18.00
TransUnet	✔			94.42	97.02	98.47	17.38
TransUnet		✔		92.34	95.91	97.87	**21.84**
TransUnet			✔	94.73	97.18	98.55	15.70
TransUnet	✔	✔		93.93	96.75	98.33	20.35
TransUnet		✔	✔	94.41	97.01	98.46	20.09
TransUnet	✔		✔	**95.82**	**97.78**	**98.86**	16.56
TransUnet	✔	✔	✔	95.73	97.72	98.83	19.59

The bold numbers indicate the best values for this metric. “✔” indicates that the module has been added.

**Table 7:** Discussion about the weight of the loss function.

Method	Dice Loss	BCE Loss	mIoU (%)	F1-Score
DSC-Net	0.1	0.9	97.29	98.66
DSC-Net	0.3	0.7	97.26	98.65
DSC-Net	0.5	0.5	97.49	98.73
DSC-Net	0.7	0.3	**97.72**	**98.83**
DSC-Net	0.9	0.1	97.09	98.51

The bold numbers indicate the best values for this metric.

## Data Availability

The datasets generated and analyzed during this study are not publicly accessible due to privacy protection measures for individuals depicted in real-life scenarios. However, they can be provided by the corresponding author upon reasonable request.
